# Histological Findings of Resected Tracheal Ring in SARS-CoV-2-Positive and -Negative Tracheostomized Patients

**DOI:** 10.3390/life14121655

**Published:** 2024-12-12

**Authors:** Nieves Mata-Castro, Raúl Castañeda-Vozmediano, Cristian Perna, Carlos Prada Puentes, Lorena Sanz López

**Affiliations:** 1 Department of ENT, 12 de Octubre University Hospital, 28041 Madrid, Spain; nmata@cirujanoscyc.com; 2 School of Medicine, Universidad Francisco de Vitoria, 28223 Madrid, Spain; raul.castaneda@ufv.es; 3 Department of Pathology, Ramon y Cajal Hospital, 28034 Madrid, Spain; cperna@yahoo.es; 4 RYCIS, School of Medicine Universidad de Alcala, 28801 Madrid, Spain; 5 Department of Pathological Anatomy, Torrejón University Hospital, 28850 Madrid, Spain; cprada@torrejonsalud.com; 6 Department of ENT, Torrejón University Hospital, Universidad Francisco de Vitoria, 28850 Madrid, Spain

**Keywords:** tracheostomy, COVID-19, SARS-CoV-2, histopathology

## Abstract

Introduction: The aim of this study was to compare the histopathological findings in the resected tracheal ring of tracheotomized critically ill patients with or without severe SARS-CoV-2 infection. Material and Methods: This is a prospective case–control study. The data collection period was between May 2020 and 2022. Eighty tracheostomies were performed on patients with long intubation, and the resected tracheal ring was examined by standard microscopy. Forty consecutive tracheotomies were carried out in COVID-19-positive and -negative patients. Results: The mean age was 67.1 (6.9 SD) years in the COVID-19 group and 67.8 (9.6 SD) in the control group (*p* = 0.3). The number of men in each group was 30 (75.0%) versus 27 (67.5%), respectively (*p* = 0.5). No relevant histological alterations were found in 82.5% of samples. Chronic subepithelial inflammation was found in 13.8% of cases. Two cases presented with vasculitis (2.5%), and one case presented with thrombotic microangiopathy (1.2%), all of them in the COVID-19 group. We found no statistically significant dependence between relevant histologic findings versus no alterations (*X*^2^ = 0.779, *p*= 0.377) and no significant risk indices (*RR* = 1.8, *OR* = 2.032, *PAR* = 44%). Conclusion: There is no evidence of increased risk of histopathological findings in the resected tracheal ring of patients with long intubation and COVID-19 disease.

## 1. Introduction

The main organs affected by severe acute respiratory syndrome coronavirus 2 (SARS-CoV-2) disease (COVID-19) are lungs where tissue hypoxia occurs, causing diffuse alveolar damage with severe capillary congestion, as seen on post-mortem examinations [1,2,3,4]. Alveolocapillary microthrombi and specific vascular angiogenesis are more frequent in the lungs of patients with COVID-19 than in those with influenza A (H1N1) [5]. In parallel, post-mortem studies have also shown the presence of tracheal injuries. The autopsy findings of 21 patients with COVID-19 revealed severe mucous tracheitis in a third of them, also describing lymphoid infiltrates in the trachea [1].

Autophagosomes with viral aggregates may be present in tracheal epithelial cells and within extracellular mucus in the tracheal lumen [6]. In another post-mortem study with 14 cases, mild inflammatory changes in the submucosa, edema with small lymphocytic aggregates, and focal acute tracheitis were observed [2]. The in situ expression of SARS-CoV-2 was detected in tracheal sections in 4 of 7 autopsies using reverse transcriptase–polymerase chain reaction (RT-PCR) [7]. In a multi-institutional study involving 68 autopsies, small white aphthous ulcers were described in the SARS-CoV-2 inflammation of the trachea and virus was observed in the airway epithelium by RNAscope^®^ technology [8].

Similar findings could be found in the trachea of the COVID-19 tracheostomized patients. The aim of this study is to show the histological findings in the tracheal tissue of COVID-19 tracheostomized patients and to determine if there is an association between these findings and SARS-CoV-2 infection.

## 2. Materials and Methods

**Figure 1:**
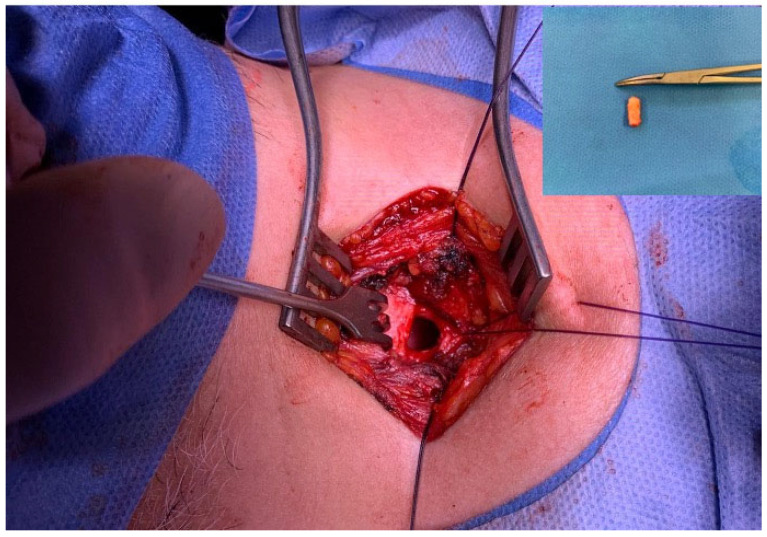
Intraoperative images of tracheostomy with resection of tracheal ring.

The data collection period was between May 2020 and May 2022. We examined 80 tracheal samples obtained during tracheostomy (Figure 1) from patients admitted to the intensive care unit undergoing long-term endotracheal intubation. The tracheal tissues were studied using standard microscopy techniques.

The diagnosis of positive COVID-19 was confirmed by a nasal antigen test and polymerase chain reaction. None of these patients were vaccinated against SARS-CoV-2 infection.

The following variables were collected: sex, age, COVID-19 disease (yes/no), comorbidities (yes/no), histopathological findings (yes/no), Positive End-Expiratory Pressure (PEEP), and the ratio of the partial pressure of oxygen in arterial blood (PaO_2_) to the inspired oxygen fraction (FiO_2_) (PAFI) at the time of intubation and at the day of the tracheostomy.

The characteristics of the patients were described and compared between the SARS-CoV-2-positive group and the SARS-CoV-2-negative group to study the homogeneity and comparability of both samples. All patients included in the COVID-19 group did not have any other associated illness besides pneumonia. The hypothesis of independence between suffering SARS-CoV-2 infection and presenting relevant histologic findings was also studied by the Chi-square test, Fisher’s exact test, and Cramer V as the association measurement index. Patients in the control group had various comorbidities, but it was found that both groups are a homogeneous and normalized sample. Finally, some risk indices such as the Relative Risk (RR) index, the odds ratio (OR), or the Percentage of Attributable Risk (PAR) were estimated. Differences in the quantitative variables between the different groups compared were performed using the Mann–Whitney U test and Wilcoxon’s R test (r) as the size effect index.

The type I error probability used was 5%. Statistical analyses on the data collected were carried out using R software version 4.4.1 (R Core Team, 2020) [9].

## 3. Results

### 3.1. Characteristics and Differences Between Patients with and Without SARS-CoV-2 Infection

The mean age (SD) was 67.1 (6.9) years in the SARS-CoV-2-positive group and 67.8 (9.6) years in the SARS-CoV-2-negative group (*p* = 0.3). The number of men in each group was 30 (75.0%) in the first group versus 27 (67.5%) in the second group, respectively (*p* = 0.5).

The most frequent diagnosis presented by the COVID-19 group patients was pneumonia. Patients without COVID-19 disease (control group) presented with diagnoses that also required prolonged orotracheal intubation, including stroke, multiorgan failure, pancreatitis, cerebral hemorrhage, complication of cardiac surgery, head trauma, abdominal abscesses, pneumonia, status epilepticus, and pneumomediastinum.

There appeared to be no differences (*p* > 0.05) between the SARS-CoV-2-positive group and the SARS-CoV-2-negative group in the values and percentages of days until tracheostomy, PEEP at the day of tracheostomy, PAFI at the day of tracheostomy, and comorbidities (Table 1).

However, the following variables appeared to be statistically unbalanced between the groups and of moderate-to-high magnitude: PEEP at the day of intubation (*p* < 0.001, R = 0.671) and PAFI at the day of intubation (*p* = 0.041, r = 0.318)

### 3.2. Histological Findings

Altered histology was found in 14 cases (17.5%). No relevant histological alterations were found in 82.5% of samples (*N* = 66).

Chronic subepithelial inflammation was found in 13.8% of cases (*N* = 11), and these cases were all from the samples. Two cases presented with vasculitis (2.5%), and one case presented with thrombotic microangiopathy and chronic inflammation (1.2%); all cases were in the COVID-19 group (Table 2).

**Figure 2:**
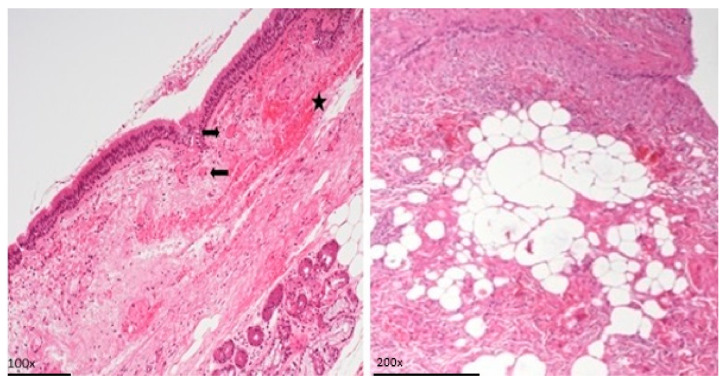
Mucosa and chorion of trachea with presence of hematic extravasation (star) and one that is fine. Caliber vessels with the lumen occupied by fibrin (arrows). The representative sample was taken from a SARS-CoV-2-positive patient. /Image of chronic subepithelial inflammation in a SARS-CoV-2-negative patient.

**Figure 3:**
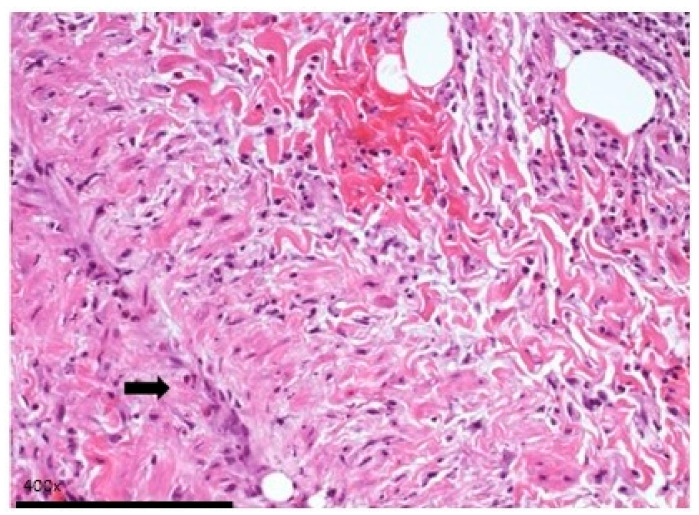
Large magnification of anterior photomicrograph. Images of a vein with venulitis (arrow). Reactive endothelial cells and mixed subendothelial and parietal inflammatory infiltrate.

The most prominent histological finding was subepithelial chronic inflammation (Figure 2) present in 13.8% of samples. Hematic extravasation and inflammation with lymphocytes, histiocytes, neutrophils, and eosinophils are observed. Endothelial cells appear reactive, and there is a mixed subendothelial and inflammatory infiltrate. Thrombotic microangiopathy was documented in one ring, and areas of hematic extravasation and vasculitis were present in one case (Figure 3).

We found no statistically significant association between relevant histologic findings and SARS-CoV-2 infection (*X*^2^ = 0.779, *p* = 0.377; *Fisher’s exact test*: *p* = 0.378; *Cramer V* = 0.132, 95%CI = 0.000–0.351).

In addition, the risk indices obtained contemplated a value of 1 in the estimated 95% confidence intervals (*RR* = 1.8, 95% CI = 0.66–4.9; *OR* = 2.032, 95% CI = 0. 614–6.716; *PAR* = 44%, 95% CI = −51.2–79.6). Therefore, although the risk of suffering alterations in the SARS-CoV-2-positive group seemed to be descriptively higher than in the SARS-CoV-2-negative group, we could not affirm that SARS-CoV-2 infection represented a significantly higher risk (Table 3).

On the other hand, none of the variables mentioned in the previous section seem to be associated with the appearance of relevant histological findings (Table 4), except for PAFI at the day of tracheostomy (*p* < 0.041, r = 0.229). The group with no relevant histological findings had a mean PAFI at the day of tracheostomy score of 193.0 (45.2), significantly higher than the group with an altered histology (160.2 (50.2)).

## 4. Discussion

In the Community of Madrid, Spain, the first wave of SARS-CoV-2 infection occurred in March 2020. The first 19 tracheotomies performed on patients with COVID-19 pneumonia and prolonged intubation were excluded, as they were conducted in an emergency phase with personal protective equipment and using the standard tracheostomy technique, where the resected tracheal ring was not sent for pathological examination [10]. The second challenge of this study was to collect enough patients who underwent tracheostomy due to prolonged intubation but with a diagnosis other than SARS-CoV-2 pneumonia to compare both groups.

This study was therefore conducted in an exceptional epidemiological situation, without information on the risks associated with performing the tracheotomy and the expected findings in the trachea.

Now, we know that our study expands the literature by documenting six cases of altered histology in tracheal samples obtained in vivo during tracheostomy performed on patients with COVID-19 disease and oral prolonged intubation. Since ulceration and edema of the subglottis extending beyond the third tracheal ring have been described in COVID-19 cases, rendering extubation impossible [10,11], we aimed to determine whether there were any pathological findings in the resected ring during tracheostomy.

We found a case report in the literature that describes histopathological findings from a surgical specimen after tracheal resection for post-tracheostomy stenosis in a COVID-19 patient. The histologic findings of the tracheal sample included lymphomonocytic perivascular inflammatory infiltrate and giant cell granulomas, coagulative necrosis of the submucosal tissue, and neoangiogenesis [12]. Similarly, in our study, we report the presence of lymphocytic and plasma cells inflammatory infiltrate, hematic extravasation, and fine-caliber vessels with lumens occupied by fibrin, as well as venulitis in the tracheal ring of tracheostomized COVID-19 patients.

These inflammatory findings may provide a histological explanation for the macroscopic changes observed by the surgeon during tracheostomy. Our clinical observations align with studies suggesting that airway vasculitis phenomena may contribute to the increased incidence of tracheal injuries [13]. Additionally, inflammation was also noted in another study that evaluated, by histology and immunohistochemistry, the fibrotic tissue resected after a subglottic stenosis post-tracheostomy in a severe ill COVID-19 patient, which revealed a high localized density of immunoglobulin G4 (IgG4)-secreting plasma cells [14].

The interest of our study is twofold: first, we aimed to document the morphological changes associated with SARS-CoV-2-related tracheitis in “in vivo” samples, avoiding post-mortem detachment of epithelial cells, and secondly, to explain the relationship between oxygenation and the presence of histological findings in patients with COVID-19 who underwent intubation and tracheotomy.

In 2022, Ward et al. conducted a study comparing tracheal rings in COVID-19 and non-COVID-19 tracheostomized patients, evaluating 38 tracheal rings excised at tracheostomy from long-term intubated COVID-19 patients and 5 from long-term intubated non-COVID-19 patients. They also underwent histological examination on four tracheal autopsy samples from COVID-19 patients who died without undergoing prolonged mechanical ventilation. The histological findings were similar between mechanically ventilated COVID-19-positive and -negative patients [15].

Similarly, our study concludes that there is no increased risk of presenting histological abnormalities in the presence of SARS-CoV-2 infection. Findings associated with chronic inflammation may also be present in patients undergoing tracheostomy due to prolonged intubation without SARS-CoV-2 infection.

Analyzing in depth the characteristics of the groups in our study, we observed differences between them in terms of comorbidity, severity of the disease, and oxygenation.

The PEEP levels at the time of intubation were higher in the COVID-19 group, suggesting more severe respiratory involvement. An elevated PEEP could generate changes in the tracheal structure due to prolonged ventilatory support and pressure on the tissues, but no differences were found between the groups with/without findings.

The PaO_2_/FiO_2_ ratio (PAFI) was lower in the COVID-19 group at the time of intubation, indicating the compromise in the oxygenation of these patients, but these differences evened out by the time of tracheostomy, indicating the time of best oxygenation and stabilization of the patient to perform the tracheostomy.

PAFI was higher at the time of tracheostomy in those without histological findings, suggesting that better oxygenation might be linked to the absence of significant tracheal pathology.

## 5. Conclusions

The histological examination of the tracheal ring of tracheostomized SARS-CoV-2-positive patients presents subepithelial chronic inflammation, thrombotic microangiopathy, and vasculitis phenomena. There is no statistically significant association between relevant histologic findings and SARS-CoV-2 infection. There is no evidence of an increased risk of histopathological findings in the resected tracheal ring of tracheostomized COVID-19 patients. The oxygenation status at the time of the tracheostomy is linked to the presence or absence of pathological findings in the tracheal ring.

## Figures and Tables

**Table 1:** Characteristics and differences between the group with and without SARS-CoV-2.

	Overall, *N* = 80 ^1^	SARS-CoV-2-Negative, *N* = 40 ^1^	SARS-CoV-2-Positive, *N* = 40 ^1^	*p*-Value	Effect Size
SEX				0.5 ^2^	0.055
Male	57 (71.3%)	27 (67.5%)	30 (75.0%)		
Female	23 (28.8%)	13 (32.5%)	10 (25.0%)		
AGE				0.3 ^3^	0.116
Mean (SD)	67.4 (8.3)	67.8 (9.6)	67.1 (6.9)		
Median [25–75%]	69.0 [63.0–73.0]	71.0 [59.8–75.3]	68.0 [63.0–70.5]		
Days until tracheostomy				0.091^3^	0.190
Mean (SD)	17.2 (4.7)	18.5 (4.2)	16.0 (4.9)		
Median [25–75%]	17.0 [15.0–20.0]	18.0 [15.8–20.3]	16.0 [12.5–19.0]		
PEEP at intubation				**<0.001 ^3^**	0.671
Mean (SD)	10.4 (2.6)	**8.8 (1.8)**	**12.2 (2.1)**		
Median [25–75%]	10.0 [8.0–12.0]	8.0 [7.0–10.3]	12.0 [10.0–14.0]		
PEEP at tracheostomy				0.5 ^3^	0.084
Mean (SD)	9.6 (2.1)	9.4 (2.0)	9.8 (2.2)		
Median [25–75%]	10.0 [8.0–11.0]	9.5 [8.0–11.0]	10.0 [8.0–12.0]		
PAFI at intubation				**0.041 ^3^**	0.318
Mean (SD)	156.1 (86.7)	**226.0 (97.9)**	**146.6 (82.0)**		
Median [25–75%]	136.0 [100.0–183.3]	180.0 [180.0–200.0]	120.0 [100.0–181.0]		
PAFI at tracheostomy					
Mean (SD)	187.5 (47.5)	187.5 (49.3)	187.5 (46.1)	0.9 ^3^	0.019
Median [25–75%]	190.0 [157.3–209.3]	190.0 [160.0–200.0]	190.0 [150.0–213.0]		
COMORBITIES				**<0.001 ^3^**	0.501
Mean (SD)	6.0 (5.0)	**8.3 (4.4)**	**3.6 (4.5)**		
Median [25–75%]	7.0 [0.0–11.0]	10.0 [2.5–11.0]	1.0 [0.0–8.5]		
Unknown	3	1	2		
HISTOLOGICAL FINDINGS					
normal	66 (82.3%)	35 (87.5%)	31 (77.5%)	**<0.0012**	0.427
subepithelial chronic inflammation moderate	6 (7.5%)	0 (0.0%)	6 (15.0%)		
low subepithelial chronic inflammation	5 (6.3%)	5 (12.5%)	0 (0.0%)		
subepithelial chronic inflammation + vasculitis	2 (2.5%)	0 (0.0%)	2 (5.1%)		
subepithelial chronic inflammation + microangiopathy thrombotic	1 (1.3%)	0 (0.0%)	1 (2.6%)		

^1^ n (%); mean (SD); median [IQR], ^2^ Pearson’s Chi-squared test, ^3^ Wilcoxon’s rank sum test.

**Table 2:** Histological findings in SARS-CoV-2-positive group and SARS-CoV-2-negative group.

	Without Histological Alterations	Chronic Subepithelial Inflammation	Vasculitis	Thrombotic Microangiopathy	Total	X^2^ (df)	*p*
SARS-CoV-2-negative group	35 (53.0%)	5 (45.5%)	0 (0.0%)	0 (0.0%)	40 (100%)	3.33 (3)	0.343
SARS-CoV-2-positive group	31 (47.0%)	6 (54.5%)	1 (100.0%)	2 (100.0%)	40 (100%)		
TOTAL	66 (82.5%)	11 (13.75%)	1 (1.25%)	2 (2.5%)	80 (100%)		

X^2^ = Chi-square statistic; df = degree of freedom.

**Table 3:** Odds ratio of presenting histological changes in the SARS-CoV-2-positive group and in the SARS-CoV-2-negative group.

	Relevant Findings	No Relevant Findings	Total	Prevalence x100	Odds	OR [95%CI]	X^2^ (df)	*p*
SARS-CoV-2-negative group	5	35	40	12.5	0.143	2.032 [0.614–6.716]	0.779 (1)	0.377
SARS-CoV-2-positive group	9	31	40	22.5	0.290			
TOTAL	14	66	80	17.5	0.212			

OR = odds ratio; CI = confidence interval; X^2^ = Chi-square statistic; df = degree of freedom.

**Table 4:** Characteristics and differences between groups with relevant histological results or not.

	Overall *N* = 80 ^1^	No Relevant Findings *N* = 66 ^1^	Yes Relevant Findings *N* = 14 ^1^	*p*-Value	Effect Size
SAR-CoV-2 +1/−0				0.2 ^2^	0.099
Negative	40 (50.0%)	35 (53.0%)	5 (35.7%)		
Positive	40 (50.0%)	31 (47.0%)	9 (64.3%)		
SEX				>0.9 ^3^	0.000
Male	57 (71.3%)	47 (71.2%)	10 (71.4%)		
Female	23 (28.8%)	19 (28.8%)	4 (28.6%)		
AGE				0.8 ^4^	0.030
Mean (SD)	67.4 (8.3)	67.6 (7.8)	66.8 (10.7)		
Median [25–75%]	69.0 [63.0–73.0]	69.0 [63.0–72.0]	70.5 [55.0–75.0]		
Day until tracheostomy				>0.9 ^4^	0.008
Mean (SD)	17.2 (4.7)	17.1 (4.3)	17.9 (6.4)		
Median [25–75%]	17.0 [15.0–20.0]	17.0 [15.0–20.0]	18.0 [15.0–19.0]		
PEEP at intubation				>0.9 ^4^	0.002
Mean (SD)	10.4 (2.6)	10.4 (2.7)	10.4 (1.7)		
Median [25–75%]	10.0 [8.0–12.0]	10.5 [8.0–12.0]	10.0 [9.0–12.0]		
Unknown	3	2	1		
PEEP at tracheostomy				0.9 ^4^	0.018
Mean (SD)	9.6 (2.1)	9.5 (2.2)	9.6 (1.8)		
Median [25–75%]	10.0 [8.0–11.0]	10.0 [8.0–11.0]	10.0 [8.0–11.0]		
PAFI at intubation				>0.9 ^4^	0.007
Mean (SD)	156.1 (86.7)	158.7 (91.4)	144.9 (66.7)		
Median [25–75%]	136.0 [100.0–184.0]	136.0 [100.0–181.0]	130.0 [100.0–204.0]		
PAFI at tracheostomy				**0.041 ^4^**	0.229
Mean (SD)	187.5 (47.5)	193.3 (45.2)	160.2 (50.2)		
Median [25–75%]	190.0 [156.5–209.5]	192.5 [160.0–216.0]	178.0 [143.0–190.0]		
COMORBITIES					
Mean (SD)	6.0 (5.0)	6.0 (5.0)	6.1 (5.3)	0.7 ^4^	0.038
Median [25–75%]	7.0 [0.0–11.0]	9.0 [0.0–11.0]	5.5 [0.0–11.0]		
Unknown	3	3	0		

^1^ n (%); mean (SD); median [IQR], ^2^ Pearson’s Chi-squared test, ^3^ Wilcoxon’s rank sum test, ^4^ Fisher’s exact test.

## Data Availability

No data associated with our study have been deposited into a publicly available repository.
